# Purinergic Signaling: A Common Path in the Macrophage Response against *Mycobacterium tuberculosis* and *Toxoplasma gondii*

**DOI:** 10.3389/fcimb.2017.00347

**Published:** 2017-08-07

**Authors:** Laetitia Petit-Jentreau, Ludovic Tailleux, Janine L. Coombes

**Affiliations:** ^1^Institute of Infection and Global Health, Department of Infection Biology, University of Liverpool Liverpool, United Kingdom; ^2^Mycobacterial Genetics Unit, Institut Pasteur Paris, France; ^3^Unit for Integrated Mycobacterial Pathogenomics, Institut Pasteur Paris, France

**Keywords:** purinergic agents, *Mycobacterium tuberculosis*, *Toxoplasma gondii*, ATP, macrophages, innate immunity, nucleotides

## Abstract

Immune responses are essential for the protection of the host against external dangers or infections and are normally efficient in the clearance of invading microbes. However, some intracellular pathogens have developed strategies to replicate and survive within host cells resulting in latent infection associated with strong inflammation. This excessive response can cause cell and tissue damage and lead to the release of the intracellular content, in particular the nucleotide pool, into the extracellular space. Over the last decade, new studies have implicated metabolites from the purinergic pathway in shaping the host immune response against intracellular pathogens and proved their importance in the outcome of the infection. This review aims to summarize how the immune system employs the purinergic system either to fight the pathogen, or to control collateral tissue damage. This will be achieved by focusing on the macrophage response against two intracellular pathogens, the human etiologic agent of tuberculosis, *Mycobacterium tuberculosis* and the protozoan parasite, *Toxoplasma gondii*.

## Introduction

**Figure 1:**
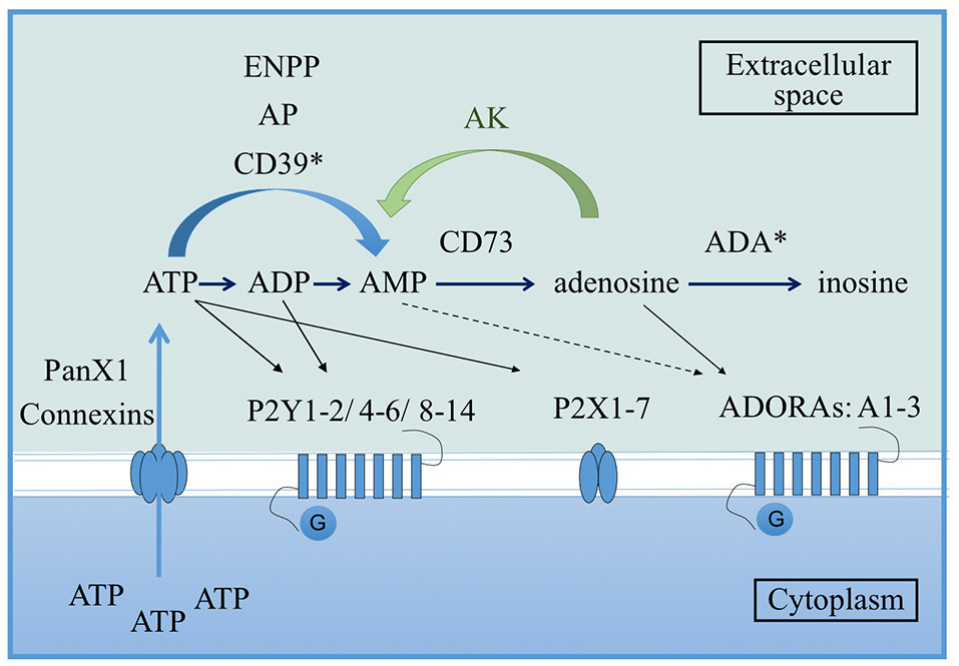
Overview of the purinergic pathway. From the cytoplasm to the extracellular space, ATP can be released via pannexin 1 (PanX1) or connexin hemichannels present on the cell membrane. Once in the extracellular environment, ATP is rapidly converted to ADP and AMP by alkaline phosphatases (AP), ectonucleotide pyrophosphatases/phosphodiesterases (ENPPs) or ecto-nucleoside triphosphate diphosphohydrolases which includes the ectonucleoside triphosphate diphosphorylase 1 (CD39). AMP is then converted to adenosine via the ecto-5′-nucleotidase CD73. Finally, adenosine is metabolized into inosine by the adenosine deaminase (ADA). Adenosine can be reversely converted into AMP by adenosine kinase (AK). In terms of receptors, ATP binds P2 receptors (either the membrane ion-channels P2X or the G protein-coupled receptors P2Y), ADP preferentially binds the P2Y receptors and adenosine binds the G protein-coupled receptors P1 also known as ADORAs. Asterisks correspond to enzymes that can be extracellular or at the surface of the cell membrane (Velasquez and Eugenin, 2014).

Purinergic nucleotides are largely known for their role as the primary energy currency of cells. However, it has been demonstrated that these nucleotides are also implicated in the modulation of the immune response (Burnstock and Boeynaems, 2014; Cekic and Linden, 2016). During inflammation, infection or after tissue injury, purines can be released into the extracellular space either passively by dying or damaged cells, or actively via pannexin or connexin hemichannels from immune cells (Beyer and Steinberg, 1991; Bao et al., 2004; Pelegrin and Surprenant, 2009; Figure 1). Extracellular ATP (eATP) is the most studied purine for its capacity to modulate the immune response. Once in the extracellular environment, eATP is rapidly metabolized to adenosine diphosphate (eADP) and adenosine monophosphate (eAMP) by alkaline phosphatases, ectonucleotide pyrophosphatases/phosphodiesterases (ENPPs) or ecto-nucleoside triphosphate diphosphohydrolases (ENTPDases) which includes the ectonucleoside triphosphate diphosphorylase 1, CD39 (Burnstock and Boeynaems, 2014). eAMP is then converted to adenosine by the ecto-5′-nucleotidase, CD73 (Burnstock and Boeynaems, 2014). Extracellular adenosine (eADO) is finally metabolized into extracellular inosine by the adenosine deaminase (ADA). In order to modulate the immune response, purine metabolites act through two families of purinergic receptors: P1 and P2 receptors (Burnstock, 2017). ATP binds to P2 receptors which are divided into ionotropic P2X and metabotropic P2Y subtypes (Ralevic and Burnstock, 1998). P1 receptors, which are also known as ADORAs receptors or A1, A2A, A2B, A3, preferentially recognize eADO (Burnstock and Boeynaems, 2014; Figure 1).

In the extracellular environment, ATP is recognized as a Damage Associated Molecular Pattern (DAMP). High concentrations of eATP alert the immune system and mediate pro-inflammatory effects. eATP induces granule release by neutrophils, T-cell activation, cytokine and chemokine secretion by macrophages, generation of reactive oxygen (ROS) or nitrogen species and dendritic cell maturation and migration (Bours et al., 2006). By contrast, eADO is best known for its immunosuppressive capacities, inhibiting neutrophil phagocytosis via the activation of A2A receptor and ROS generation by macrophages and neutrophils, improving the secretion by macrophages of wound healing and angiogenesis molecules such as, VEGF and inducing a Th2-like profile (Bours et al., 2006).

Here, we will discuss how members of this pathway can be employed by macrophages to combat intracellular pathogens or to dampen inflammation. By focusing on the response against two different pathogens: *Mycobacterium tuberculosis (M. tuberculosis)* and *Toxoplasma gondii (T. gondii)*, we will demonstrate the highly conserved mechanisms used by the purinergic pathway. Both pathogens cause major public health problems worldwide: *M. tuberculosis* is the human etiologic agent of tuberculosis (TB), while the apicomplexan parasite, *T. gondii* causes serious health problems in immunocompromised people and the developing fetus. These pathogens infect, respectively, one-third of the human population and have co-evolved with the human population for centuries (McLeod et al., 2009). Both infections share many characteristics, they can cause acute disease, or they can be latent and asymptomatic. Latent infections represent the majority of the cases after infection with either of these two pathogens. Latency could be explained by the capacity of both pathogens to survive and replicate in cells of the monocyte/macrophage lineage, and their capacity to shield them from the immune response by residing in a non-fusogenic vacuole inside the macrophage. However, in immunocompromised people, both infections manifest as acute disease. Induced pathologies are characterized by a pro-inflammatory Th1 response with a dysregulated immune response leading to a strong inflammation and collateral associated tissue damage.

During these infections, modulators are need to alert, stimulate, and regulate the immune system and nucleotides from the purinergic pathway could be one of them. In this context, these two intracellular pathogens are really good models to study the role of these purinergic mediators in the control of intracellular infections during acute phase of the disease. Here, we will discuss the recent advances in the purinergic pathway field by comparing the macrophage response against *M. tuberculosis* and *T. gondii*.

## eATP and P2X7 receptor: the killer side of the force

**Figure 2:**
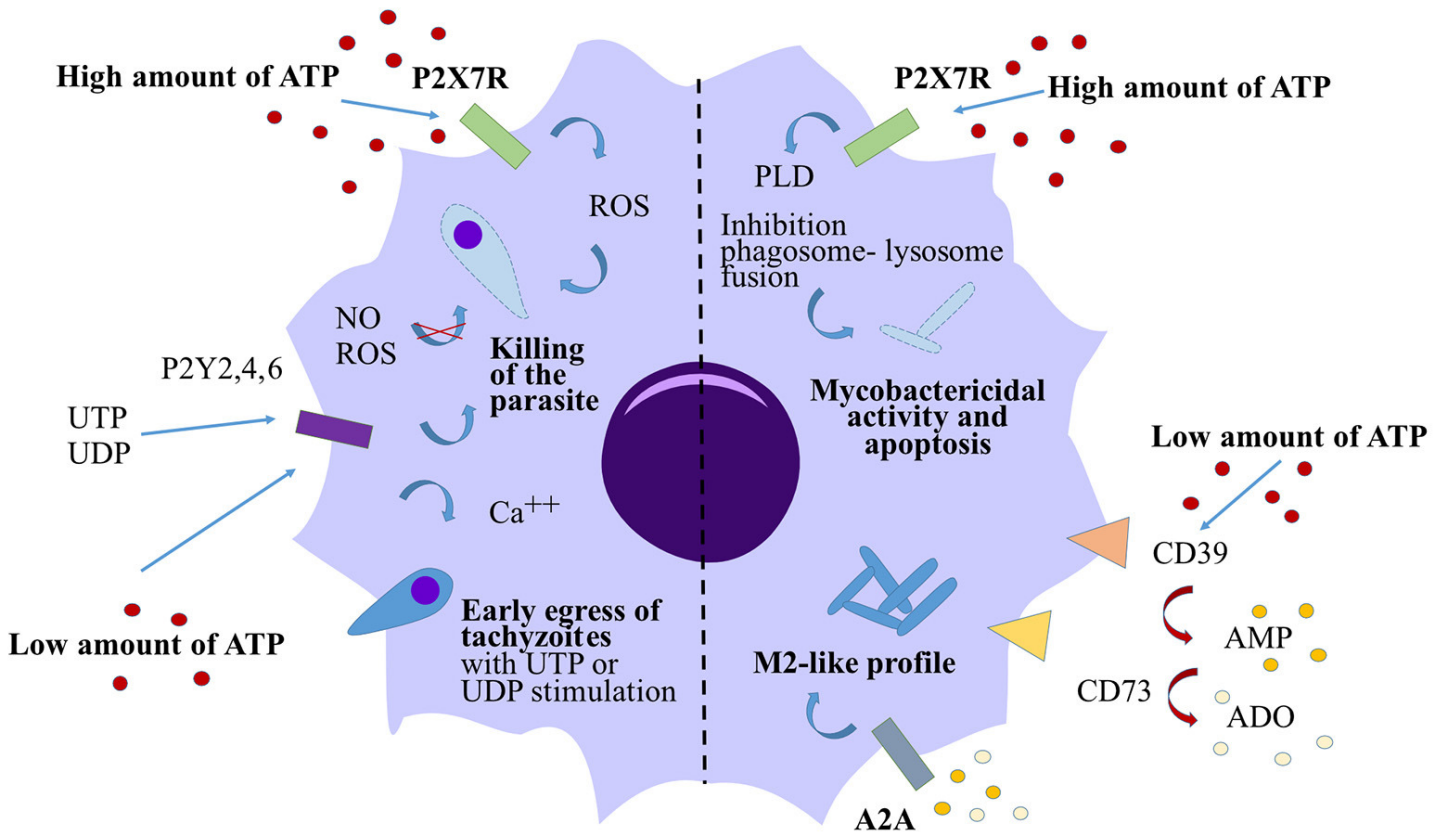
Action of eATP on macrophage response against *T. gondii* and *M. tuberculosis*. High concentrations of eATP (3–5 mM) induce the activation of P2X7R on either *T. gondii-* or *M. tuberculois-* infected macrophages and induce the killing of the pathogen in both cases. The killing of the parasite is dependent on ROS generation however the mycobactericidal activity is due to the activation of the phophalipase D (PLD). In both cases, high levels of eATP induce macrophage apoptosis. By contrast, low levels of eATP (100 μM) are rapidly converted to eAMP and eADO via CD39 and CD73. eAMP and eADO bind the adenosine receptor A2A leading to a switch in macrophage polarization toward a M2-like profile in *M. tuberculosis*-infected macrophages. Low levels of ATP control *T. gondii* infection via pyrimidinergic receptor activation without affecting the macrophage death or the production of NO or ROS. UTP or UDP stimulations induce premature egress of tachyzoites through P2Y receptors.

Cells from the macrophage/monocyte lineage represent one of first lines of defense against infection, and are the main cell targets for invasion by *M. tuberculosis* or *T. gondii*. Due to their important contribution to pathogen clearance, a number of studies have been focused on the role played by the purinergic pathway in influencing macrophage killing function. In 1994, Molloy et al. provided the first evidence for the capacity of 1 mM eATP to induce mycobacterial killing by human monocytes (Molloy et al., 1994). Lammas et al. corroborated these observations and proved that the activation of the eATP specific P2X7 receptor (P2X7R) with 3 mM ATP for 30 min was necessary to induce apoptosis of BCG-infected macrophages and killing of the bacteria, both independently of ROI/RNI (Lammas et al., 1997). Further studies demonstrated that this mycobacterial killing mechanism was dependent either on phospholipase D activation (Kusner and Adams, 2000; Fairbairn et al., 2001) or followed the apoptosis of infected macrophages (Placido et al., 2006). Similarly, using a *T. gondii* infection model, Correa et al. demonstrated that stimulation of murine macrophages with 3 mM ATP for 30 min triggered the elimination of the parasite in a P2X7R dependent manner (Corrêa et al., 2010). This killing was associated with an increase in production of ROS in infected macrophages after ATP stimulation. Lees et al. confirmed these murine data and provided evidence that P2X7R mediated killing of *T. gondii* is independent of nitric oxide (NO) secretion but is associated with host cell apoptosis (Lees et al., 2010). Overall, these data suggest a conserve mechanism for ATP in the control of bacterial or parasitic infection. High concentrations of ATP activate the P2X7R and can induce the apoptosis of infected cells leading to the death of the pathogen (Figure 2).

The role of P2X7R in the control of infection is also suggested by studies focusing on P2X7R polymorphisms in the human population. The P2X7R gene is highly polymorphic and different non-synonymous single nucleotide polymorphisms (SNPs) have been described (Sluyter and Stokes, 2011). The 1513A>C polymorphism, conferring a loss of function of the receptor, has been extensively studied in control of intracellular pathogen infections. Lees et al. (2010) demonstrated that macrophages from a patient with the 1513A>C polymorphism would be less effective at killing *T. gondii* tachyzoites after ATP exposure than macrophages from people with the 1513A wild-type (WT) allele. Another study provided evidence for an association between loss of function of P2X7R caused by another SNP, 1068 T>C, and retinal toxoplasmosis (Jamieson et al., 2010). These publications demonstrated an important role of the P2X7R in the determination of resistance or susceptibility to acquired *T. gondii* infection (Jamieson et al., 2010). The association of P2X7R gene polymorphisms with resistance to TB in humans was also studied in different meta-analyses where the 1513A>C loss of function SNP was associated with susceptibility to pulmonary (Sharma et al., 2010; Areeshi et al., 2013; Wu et al., 2015; Shamsi et al., 2016) and extra-pulmonary TB (Sharma et al., 2010; Ben-Selma et al., 2011; Singla et al., 2012). This susceptibility was due to the reduction of the capacity of macrophages to kill the bacillus (Fernando et al., 2007). Moreover, TB patients carrying this 1513A>C allele presented an increase number of bacilli in sputum than people with the WT allele, strongly implicating this particular SNP in the modulation of mycobacterial immunity (Wu et al., 2015). Others alleles for the P2X7R gene, such as, P2X7R-762C and P2X7R-1229T were also associated with a high risk of developing TB in the worldwide population (Sambasivan et al., 2010; Singla et al., 2012; Wu et al., 2015). At a global level, it has been described that the expression of P2X7R mRNA was higher in the PBMCs from TB patients compared to control patients (Franco-Martínez et al., 2006).

The role of P2X7R in human diseases was clearly demonstrated but its implications in mouse models of infection are less well characterized. In an oral model of toxoplasmic ileitis with 10 cysts of ME49 strain, Miller et al. showed no differences in parasite loads between P2X7R knock-out (KO) mice and WT mice but animals deficient for the receptor exhibit higher degrees of intestinal pathology associated with elevated ileal concentrations of pro-inflammatory cytokines (Miller et al., 2015). By contrast, Huang et al. demonstrated the high susceptibility to P2X7R KO mice to *T. gondii* infection in an oral infection with high numbers of tachyzoites from RH or *Prugniaud* strain (Huang et al., 2016). This study showed a higher *T. gondii* tachyzoite burden in the ileum of orally-infected P2X7R KO mice 5 days post-infection compared to the WT mice. This elevated parasite burden was accompanied by an impaired recruitment of CD103^+^ dendritic cells to the small intestine in the knockout infected animals compared to WT mice due to the reduced chemokine production by P2X7R KO epithelial cells (Huang et al., 2016). The high susceptibility of P2X7R KO mice to *T. gondii* compared to their WT counterpart were also confirmed in a model of acute toxoplasmosis. P2X7R KO mice succumbed faster to the infection, lost more weight, had higher parasitic loads and produced less pro-inflammatory cytokines than WT mice after intra peritoneal infections with two different strains RH or ME49 (Miller et al., 2011; Corrêa et al., 2016).

In the TB infection mouse model, the susceptibility of the P2X7R deficient mice to the infection is critically dependent on the mycobacterial dose and strains used. An initial study indicated that P2X7R was not involved in the control of pulmonary TB in mice infected with a low dose of *M. tuberculosis* Erdman strain using the aerosol route (Myers et al., 2005). By contrast, a study demonstrated that mice lacking P2X7R had a higher mycobacterial burden in the lungs compared to WT animals infected intravenously with a high dose of the common laboratory strain H37Rv (Santos et al., 2013). However, two recent papers suggested a role for the P2X7R in Bone Marrow-derived cells in aggressive forms of tuberculosis only (Amaral et al., 2014; Bomfim et al., 2017). In these studies, mice deficient for the P2X7R infected with virulent strains (Beijing1471 or MP287/03), but not with H37Rv strain, were less susceptible than WT mice (Amaral et al., 2014). Infected P2X7R KO mice or chimeric mice adoptively transferred with P2X7R KO bone marrow cells presented a decrease in the bacterial load and in the gross pathology in the lungs compared to their counterparts (Amaral et al., 2014; Bomfim et al., 2017). Moreover, the authors showed that hypervirulent mycobacteria induce macrophage necrosis in a P2X7R-dependent mechanism (Amaral et al., 2014). This deleterious role of P2X7R in severe TB can be explained by a vicious cycle where high concentrations of eATP is released by necrotic cells and lead to lung damage and bacillus dissemination.

## eATP: A role outside pathogen killing?

Dependently of the concentration, eATP may also play opposite roles in the outcome of the infection. Activation of the P2X7R with very high concentrations of ATP (3–5 mM) were clearly necessary to decrease the bacterial load or parasitic burden *in vitro* and these concentrations of ATP are determinant in tissue pathology *in vivo*. However, these high concentrations of ATP were shown to induce the apoptosis of either *T. gondii*-infected murine macrophages or *M. tuberculosis*-infected macrophages (Lees et al., 2010; Figure 2). Canaday et al. (2002) demonstrated that human monocytes exposed to 1–3 mM ATP were directly lysed after 4 h of stimulation whereas 300 μM ATP didn't cause any cell death. In the same study, they suggested that T cells or bystander myeloid cells release very low concentrations of ATP after degranulation and they proved that this bystander eATP was not sufficient to kill intracellular BCG by monocytes. However, in *T. gondii*-infected murine macrophages, 100 μM ATP was sufficient to reduce the parasite burden in a P2Y receptor dependent manner, without affecting the macrophage death and their capacity to generate NO or ROS production (Moreira-Souza et al., 2015; Figure 2). By contrast, our group recently demonstrated that stimulation with 100 μM eATP did not trigger *M. tuberculosis* elimination by human monocyte-derived macrophages (Dubois-Colas et al., 2014). Moreover, stimulation with 100 μM ATP induced a switch in the macrophage polarization into an M2 phenotype. *M. tuberculosis*-infected cells and treated with ATP, secreted less pro-inflammatory cytokines but were able to secrete more cytokines implicated in wound healing. After 18 h of stimulation with 100 μM ATP, eAMP was the main metabolite found by mass spectrometry in the supernatants of *M. tuberculosis*-infected macrophages. In this study, eATP degradation into eAMP or eADO was necessary to induce the M2-like profile both acting through A2A receptor activation (Figure 2). In that model, this concentration of ATP associated with its degradation may limit tissue damage but may also favor bacterial immune escape.

## The forgotten role of metabolites from eATP degradation

Over last decade, knock-out mouse models for enzymes involved in eATP degradation have been used to determine the role of the different purinergic nucleotides in the immune response against intracellular pathogens. CD39 is the first enzyme involved in ATP degradation, hydrolyzing eATP into eAMP (Figure 1). The role of this ectonucleotidase has been poorly explored in infectious diseases but a population of CD4^+^CD25^+^CD39^+^ T cells with regulatory properties were identified from blood of patients with active TB (Chiacchio et al., 2009). More recently, a study described a functional role for CD39 in the suppressive action of a population of *Mycobacterium bovis*-activated CD8 T cells (Boer et al., 2013). These data suggested that CD39 could be a new marker for the human regulatory CD4 or CD8 T cell populations (Miller et al., 2011; Boer et al., 2013).

In contrast to CD39, the role of the ecto-5′-nucleotidase CD73 in the outcome of intracellular infections *in vivo* has been better characterized. CD73 is involved in the conversion of eAMP into eADO (Figure 1). CD73 is a glycosylphosphatidylinositol-linked surface protein expressed by the majority of immune cells but is not highly expressed by macrophages (Antonioli et al., 2013; Dubois-Colas et al., 2014). In the context of infection, the majority of studies have been carried out using CD73 KO mice. Mahamed et al. provided the first evidence of a role for CD73 in *Toxoplasma gondii* infection. After oral infection with *T. gondii*, mice deficient for CD73 were more resistant to chronic infection, displaying decreased cyst burden in the brain compared to wild-type animals (Mahamed et al., 2012). The same authors described recently, that, contrary to chronic disease, mice deficient in CD73 were highly susceptible to acute toxoplasmosis due to damage mediated by the immune system (Mahamed et al., 2015). Thus, CD73 seems to play a role in limiting tissue damage during acute infection with *T. gondii*. This was also supported by the downregulation of CD73 expression in the small intestine during lethal ileitis triggered by oral infection of C57BL/6 mice, leading to an impaired capacity to produce eADO (Francois et al., 2015). By contrast, CD73 was not involved in the protection of *M. tuberculosis*-infected mice either at early stage of the infection or after 2 months of infection (Petit-Jentreau et al., 2015). Although *M. tuberculosis*-infected mice lacking the receptor were not more susceptible to TB than their WT counterpart, they presented a higher inflammation in the lungs due to the absence of eADO (Petit-Jentreau et al., 2015). The lungs of *M. tuberculosis*-infected CD73 KO showed a higher neutrophil recruitment at early stage of the infection compared to WT mice (Petit-Jentreau et al., 2015). The ectonucleotidase CD73 can also prevent the early recruitment of neutrophils on the site of the infection after *T. gondii* invasion limiting the inflammation (Mahamed et al., 2015). This common observation was also observed during septic choc (Haskó et al., 2011) and acute lung injury induced by lipopolysaccharide injections (Reutershan et al., 2009) where eADO can dampen the inflammation.

eADO is not the final metabolite of the purinergic pathway: it is then converted into its less functional metabolite eInosine by enzymes including adenosine-deaminase (ADA; Figure 1). During TB, eADA is present at a high concentration in the pleural liquid of infected patients (Dimakou et al., 2009) and measurement of eADA enzymatic activity in the lungs of infected patients could serve as a marker for TB infection (Blake and Berman, 1982). During toxoplasmosis, ADA activity is increased in rat T cells infected by RH *T. gondii* compared to non-infected rat (Tonin et al., 2013) but a recent study showed that eADA activity was differently modulated on the brain of *T. gondii* infected mice depending on the strain and the stage of the infection (Tonin et al., 2014). They demonstrated a decrease of eADA activity in the brain after acute infection with RH strain but an increase of the activity in the brain during the chronic phase of the infection with the cystogenic strain ME-49 supporting the idea that adenosine is necessary to decrease the inflammation.

## Summary

From parasitic to mycobacterial infections, this review underlines the very conserved and important role played by the purinergic pathway in the control of intracellular pathogens by the immune system. In this context, the ATP-mediated killing associated with activation of the P2X7R is necessary to avoid the replication of either *T. gondii* or *M. tuberculosis in vitro* and loss of function of the P2X7R in the human population by SNP in the P2X7R allele induces an higher susceptibility in the outcome of the two diseases. Adenosine generated via CD73 activity is also necessary to dampen the inflammation in acute models of both infections.

However, the action of the purinergic pathway should not solely be considered as the pro-inflammatory functions of eATP on one side, and the immunosuppressive properties of eADO (Cekic and Linden, 2016) on the other. With the recently described role played by lower concentrations of ATP (100 μM) and metabolites from ATP degradation (in particular eAMP) in limiting tissue damage during *M. tuberculosis* infection, new studies are now necessary to better understand the regulation of this balance between pro- and anti-inflammatory effects. In this review, we have discussed the complexity of this pathway where depending on the concentration used, the receptor or the enzyme involved, metabolites from the purinergic pathway can generate opposite effects. Members of the purinergic pathway represent now promising curative strategy for intracellular infections (Soares-Bezerra et al., 2015).

## Author contributions

LP-J wrote the paper with input from LT and JLC. All authors revised the manuscript, and approved it for publication.

### Conflict of interest statement

The authors declare that the research was conducted in the absence of any commercial or financial relationships that could be construed as a potential conflict of interest.
